# Anatomical cardiac and electrocardiographic axes correlate in both upright and supine positions: an upright/supine CT study

**DOI:** 10.1038/s41598-023-45528-y

**Published:** 2023-10-24

**Authors:** Togo Norimatsu, Takehiro Nakahara, Yoshitake Yamada, Yoichi Yokoyama, Minoru Yamada, Keiichi Narita, Masahiro Jinzaki

**Affiliations:** 1 Department of Radiology, Keio University School of Medicine, Shinanomachi 35, Shinjyuku, Tokyo, 160-8582 Japan; 2 Department of Vascular Surgery, Sakakibara Heart Institute, Tokyo, Japan

**Keywords:** Cardiovascular biology, Cardiovascular biology, Anatomy

## Abstract

The correlation between the anatomical cardiac and electrocardiographic axes has been discussed for several years. Using upright computed tomography, this study aimed to reveal the relationship between the anatomical cardiac and electrocardiographic axes in both the supine and upright positions. Upright CT and standard supine CT were performed for healthy volunteers, following electrocardiography in both upright and supine position. On CT images, the coordinates of apex, the center of aortic valve (AV) and mitral valve (MV) were recorded, and the vectors and angles were calculated. Subcutaneous and visceral fat volume were semi-automatically calculated in a workstation. From a total 190 volunteers, 41 males were performed electrocardiography and included for this study. The QRS and anatomical axes (AV-apex and MV-apex axis) were significantly correlated in both supine and upright positions, while the angle of the AV-apex to Z axis was the most correlated (supine: r = − 0.54, *p* = 0.0002, upright: r = − 0.47, *p* = 0.0020). The anatomical axis moved in the dorsal and caudal directions from the supine to upright position. Multiple regression analysis revealed that the anatomical axis from the AV-apex to the Z-axis was determined according to age, body height, subcutaneous and visceral fat volumes.

## Introduction

The correlation between the electrical and anatomical axes of the heart has been discussed for several years^1–4^, as well as the effect of changes in the position of the heart on the electrical QRS axis^5^. Early investigators compared the QRS axis with the anatomical cardiac axis using chest radiography and found a correlation between them^1–4^. The development of non-invasive imaging enabled the acquisition of three-dimensional (3D) anatomy; thus, investigators were able to compare the anatomical axis on a two-dimensional (2D) plane obtained from magnetic resonance imaging (MRI) or computed tomography (CT) images^6–9^. However, these studies have yielded controversial results.

We developed an upright CT that reveals the posture of organs in the upright position^10^, and determined the effect of gravity on human anatomy employing volunteers^11–14^.

This study aimed to investigate the relationship between the anatomical cardiac and electrocardiographic QRS axes in both the supine and upright positions using upright CT.

## Results

**Figure 1:**
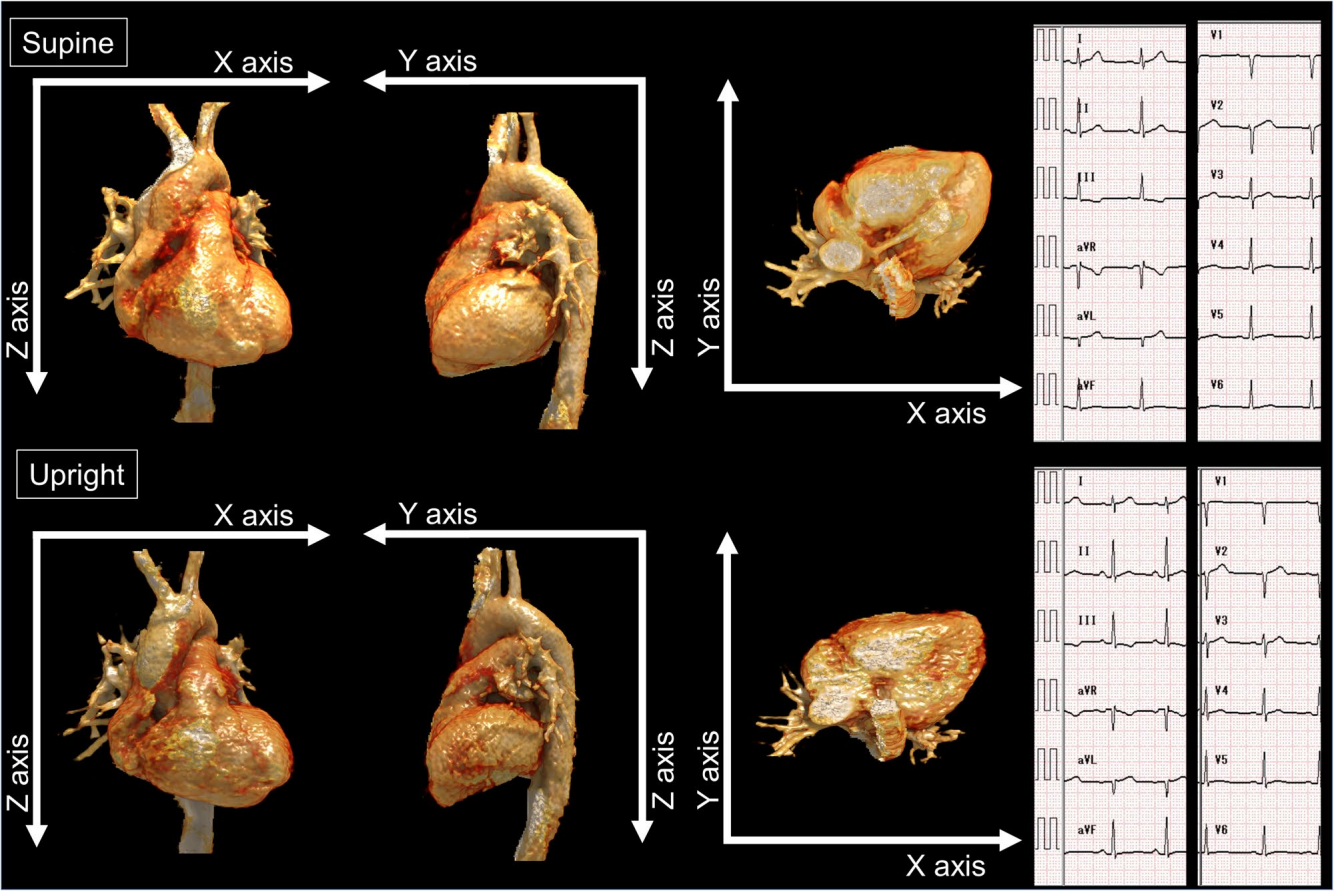
Representative case. A volume rendering image of a representative case (32-year-old male, body height of 168 cm, body weight of 71 kg, body mass index of 25.2, body circumference of 89.4 cm, subcutaneous fat volume of 184.9 cm^2^ and visceral fat volume of 92.2 cm^2^). The angles of the AV-apex to X-, Y-, and Z-axes were 48.4° versus 48.7°, 57.3° versus 61.9°, and 58.8° versus 54.2° (supine vs. upright), respectively.

Among the 190 volunteers, 41 males (48.4 ± 12.0 years) agreed and underwent ECG tests on the same day of the CT scanning (Fig. 1). Their characteristics are presented in Supplemental Table 1. Standard supine ECG diagnoses were normal, except for one volunteer (32-year-old male), who had high voltage recording and suspected left ventricular hypertrophy (LVH).

The AV- and MV-apex angles demonstrated excellent interobserver agreement against all axes (Supplemental Figs. 1–6). The ECG axis was significantly larger in the upright position than in the supine position. The 3D angle of the AV-apex to the Y-axis was significantly higher in the upright position, whereas the angle of the AV-apex to the X/Z axis was not significantly different. In the 2D analysis, the angle of the AV-apex on the XY and YZ planes changed between positions, whereas the angle on the XZ plane did not significantly change. The 3D angles of the MV-apex to all axes showed significant differences between the positions (Table 1).

**Table 1:** The difference between supine and upright.

	Supine	Upright	*p*
QRS axis (°) on ECG	72.4 ± 14.4	77.6 ± 22.8	0.0155*
Anatomical axis in 3D			
AV-apex to X axis (°)	53.3 ± 7.3	51.8 ± 7.9	0.0648
AV-apex to Y axis (°)	60.0 ± 7.1	62.4 ± 6.8	0.0036*
AV-apex to Z axis (°)	52.3 ± 10.4	51.5 ± 8.6	0.3229
MV-apex to X axis (°)	59.4 ± 6.4	56.7 ± 7.9	0.0033*
MV-apex to Y axis (°)	44.7 ± 8.0	49.6 ± 7.0	< 0.0001*
MV-apex to Z axis (°)	62.6 ± 10.6	59.5 ± 9.3	0.0033*
Anatomical axis in 2D			
AV-apex on XY plane from X (°)	39.9 ± 7.1	37.0 ± 8.4	0.0073*
AV-apex on XZ plane from X (°)	45.1 ± 11.0	45.1 ± 9.5	0.9715
AV-apex on YZ plane from Y (°)	49.9 ± 11.7	53.0 ± 9.3	0.0052*
MV-apex on XY lane from X (°)	54.2 ± 6.8	49.9 ± 8.2	< 0.0001*
MV-apex on XZ plane from X (°)	40.9 ± 13.3	42.5 ± 12.3	0.2556
MV-apex on YZ plane from Y (°)	32.6 ± 12.1	37.7 ± 10.3	< 0.0001*

(*p < 0.05).

The QRS axis correlated with anatomical angles, especially the 3D angle of the AV-apex to the Z-axis in both the supine and upright positions (supine: r = − 0.54, *p* = 0.0002; upright: r = − 0.47, *p* = 0.0020). The QRS axis was also negatively correlated with age (supine: r = − 0.43, *p* = 0.0051, upright: r = − 0.37, *p* = 0.0186), body circumference (supine: r = − 0.44, *p* = 0.0040, upright: r = − 0.53, *p* = 0.0004), subcutaneous fat volume (supine: r = − 0.35, *p* = 0.0234, upright: r = − 0.44, *p* = 0.0044), and visceral fat volume (supine: r = − 0.46, *p* = 0.0023, upright: r = − 0.49, *p* = 0.0011) in both the supine and upright positions (Table 2). Multiple regression analysis revealed that only the angle from the AV-apex to the Z-axis was associated with the QRS axis in the supine position. In the upright position, the angle of the AV-apex to the Z-axis and MV-apex to the Z-axis were associated with the QRS axis, while the angle of the AV-apex to the Z-axis had the highest association (Supplemental Table 2). The correlation between the QRS axis and age or fat volumes disappeared controlling the angle of the AV-apex to Z-axis (Supplemental Table 3).

**Table 2:** correlation between QRS axis and parameters (*p < 0.05).

Peason ‘s R (*p* value)	QRS axis in Supine	QRS axis in Upright
AV-apex to X axis (°)	0.39 (0.0113*)	0.24 (0.1366)
AV-apex to Y axis (°)	0.46 (0.0023*)	0.35 (0.0255*)
AV-apex to Z axis (°)	− 0.54 (0.0002*)	− 0.47 (0.0020*)
MV-apex to X axis (°)	0.27 (0.0935)	0.11 (0.4997)
MV-apex to Y axis (°)	0.51 (0.0007*)	0.39 (0.0119*)
MV-apex to Z axis (°)	− 0.53 (0.0004*)	− 0.40 (0.0092*)
AV-apex on XY plane from X (°)	− 0.06 (0.7219)	− 0.14 (0.3819)
AV-apex on XZ plane from X (°)	0.50 (0.0008*)	0.39 (0.0117*)
AV-apex on YZ plane from Y (°)	0.55 (0.0002*)	0.51 (0.0007*)
MV-apex on XY plane from X (°)	− 0.10 (0.5354)	− 0.07 (0.6656)
MV-apex on XZ plane from X (°)	0.49 (0.0012*)	0.32 (0.0445*)
MV-apex on YZ plane from Y (°)	0.44 (0.0037*)	0.54 (0.0002*)
Age	− 0.43 (0.0051*)	− 0.37 (0.0186*)
B.H	− 0.08 (0.6198)	− 0.25 (0.1173)
B.W	− 0.27 (0.0887)	− 0.40 (0.0099*)
BMI	− 0.19 (0.2406)	− 0.22 (0.1766)
Body circumference	− 0.44 (0.0040*)	− 0.53 (0.0004*)
Subcutaneous fat volume	− 0.35 (0.0234*)	− 0.44 (0.0044*)
Visceral fat volume	− 0.46 (0.0023*)	− 0.49 (0.0011*)

**Figure 2:**
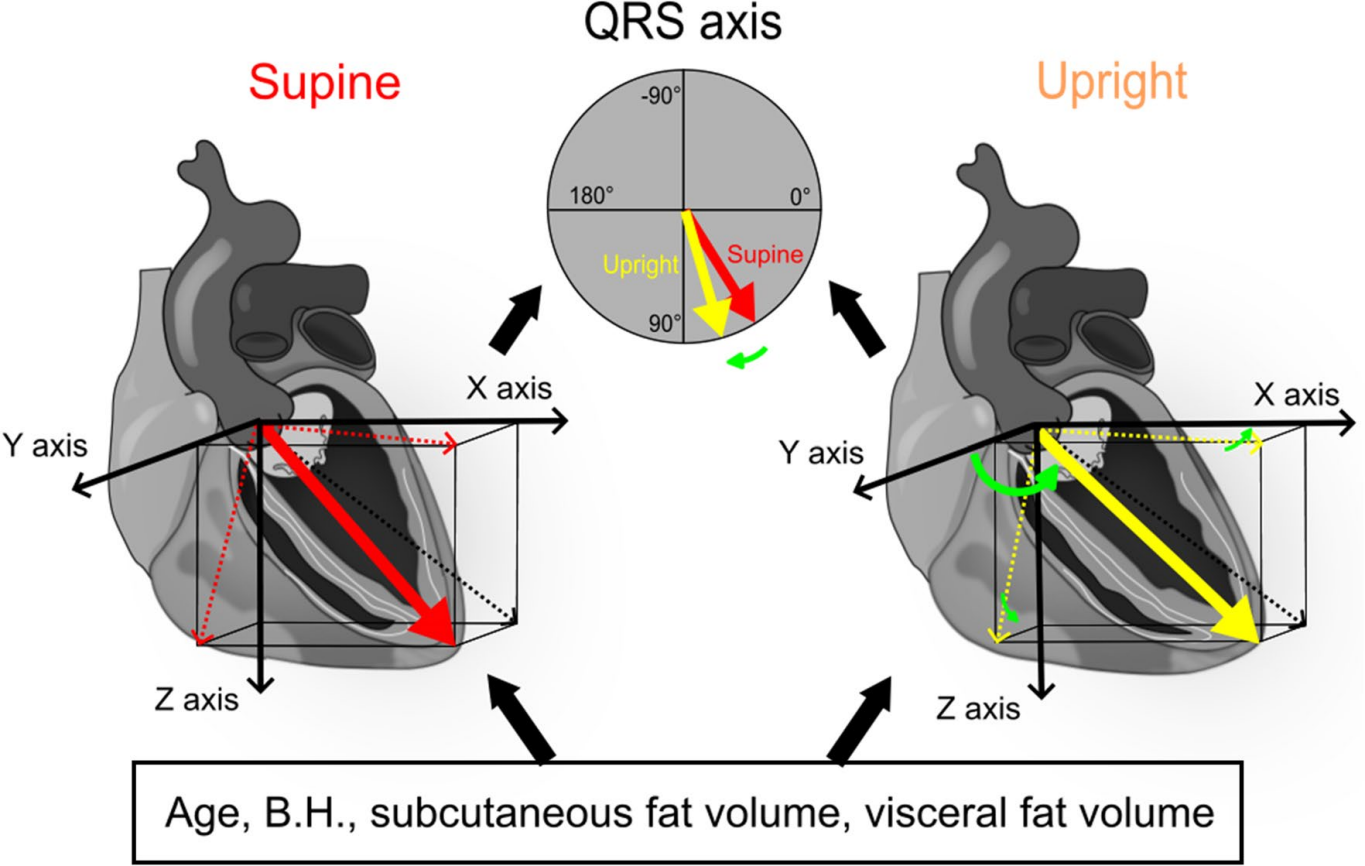
Summary of the study. The QRS axis was significantly correlated with the anatomical axis (AV-apex) in both the supine and upright positions. The anatomical axis moved in the dorsal and caudal directions from the supine to the upright position. The anatomical axis was determined based on age, body height, subcutaneous and vesicle fat volumes.

Subsequently, the correlation between the angle of the AV-apex to the Z-axis and other anatomical parameters was determined. In both the supine and upright positions, the AV-apex angle to the Z-axis was most correlated with visceral fat volume (supine: r = 0.60, *p* < 0.0001; upright: r = 0.64, *p* < 0.0001), while it also correlated with age, body height, body mass index, body circumference, and subcutaneous fat volume (Supplemental Table 4). Multiple regression analysis showed that age, body height, subcutaneous and visceral fat volumes were associated with the angle of the AV-apex to the Z axis in both the supine and upright positions (Fig. 2, Supplemental Table 5).

## Discussion

In this study, the degree of the QRS axis on the ECG in the upright position was elevated (i.e., shifted in the caudal direction) compared with that in the supine position. In the 3D analysis, the angle of the anatomical AV-apex against the Y-axis was elevated in the upright position compared to that in the supine position, while that of the X- or Z-axes showed no significant difference. In the 2D analysis, the angle of the AV-apex on the XY plane from X axis decreased, and the AV-apex on the YZ plane from Y axis increased. In other words, the AV-apex axis moved in a dorsal and caudal direction from the supine position to the upright position. Considering that only the angle to the Y-axis changed between positions, the AV-apex axis rotated around the X- and Z-axes. The angles of the anatomical MV-apex against all axes (x/y/z axes) were changed between the supine and upright positions.

The QRS axis on the ECG was most correlated with the anatomical AV-apex axis in both the supine and upright positions, while the MV-apex axis was also correlated. The anatomical AV-apex to the Z axis was most correlated with visceral fat volume, but also correlated with age, body height, body circumference, and subcutaneous fat volume.

It is generally accepted that ECG and X-ray remains the fundamental method to assess the heart^15,16^. In this study, the 3D angle of the anatomical AV-apex against the Z-axis was most correlated with the QRS axis. In 2D analysis, the correlation between the QRS axis on ECG and anatomical cardiac axis has already been discussed. In 1951, Flowler et al.^3^ showed a correlation between the QRS axis on ECG and anatomical long axis on chest roentgenography in the supine position. In 1970, Doughetry et al.^4,17,18^ investigated the relationship in both the supine and upright positions and found that the QRS axis correlated with the anatomical long axis, and the axis changed between positions with age or body build. In our study, 2D analysis showed that the AV-apex axis in the XZ plane (i.e., frontal plane) was significantly correlated with the QRS axis, which was consistent with the results of the previous studies.

A 3D modality was employed, and the anatomical axis was defined as the angle of the interventricular septum on the axial plane on CT with contrast material. The ECG axis rotation (i.e., clockwise or counterclockwise rotation) was explained from the anatomical axis in two-thirds of rotation cases^19^. Engblom et al.^7^ used MRI to define the anatomical axis as the horizontal longitudinal axis and showed a weak correlation between the anatomical and QRS axes; however, other groups showed no correlation using MRI or CT^8,9^. Although the conclusions were transverse in these CT or MRI studies^6–9^, the cardiac axis was obtained from the apex to the center of the MV annulus (i.e. MV-apex axis) in 2D cardiac long axis plane. We also assessed the cardiac axis in the 2D plane following previous studies and found a correlation between the electrical and anatomical MV-apex axes in 2D; however, we also found a higher correlation with the anatomical MV-apex axis in 3D. Furthermore, the anatomical AV-apex axis in 3D correlated the most with the QRS axis. Since the bundle branches run from the upper margin of the muscular interventricular septum, which is just below the membranous septum, to the apex^20^, the axis of the bundle branch pathway will be closer to the AV-apex axis than the MV-apex axis, leading to a greater correlation between the AV-apex and QRS axes.

In the anatomical 3D analysis, the MV-apex axis to the X/Y/Z axis was significantly changed between the supine and upright positions, while the AV-apex axis was only changed along the Y-axis. These results suggest that the movement of the AV area was relatively restricted, resulting in a greater correlation between the QRS and AV-apex axes than with the MV-apex axis. The most plausible explanation for this is the anchoring to the pericardium. The heart was surrounded by the pericardium, and the visceral pericardium was reflected near the origins of the aorta, in addition to the connection with the dorsal aspect of the sternum, tendon of the diaphragm, and short fibrosa^21,22^.

In this study, the QRS axis was negatively (i.e., leftward shift) correlated with aging and fat volumes in both supine and upright positions. Previous studies have also shown a leftward shift in the QRS axis with aging^23–25^, obesity^26–29^, or body fat, which was estimated by skin fold thickness^26^, while the prevalence of abnormal left axis deviation increased mortality^30^, suggesting that our population is comparable to the previous study population. However, a study by Doughetry et al.^18^ demonstrated that the correlation between the QRS axis and age or body build disappeared after controlling the anatomical axis. Consistent with this study, the partial correlation between the QRS axis and age or fat volumes disappeared when it was controlled by the angle of the AV-apex to the Z-axis, suggesting that these correlations were only spurious.

Therefore, the QRS axis in the ECG was prescribed by the anatomical cardiac 3D axis (the angle of the AV-apex to the Z axis), and the anatomical axis was prescribed by age, body height, subcutaneous and visceral fat volumes.

This study is a novel attempt. Thus, it has several limitations. First, since the upright CT is still a prototype machine, the number of participants that may be included in this single-center study was limited. Second, the volunteers did not receive contrast materials and ECG gated to reduce the radiation dose and side effects. Without contrast materials, the axis of the ventricular septum, in which the bundle branches run, was difficult to identify, in addition to myocardium and intracardiac cavity. Therefore, we estimated the AV-apex and MV-apex axes. This might affect the identification of 3D coordinate points; however, interobserver viability showed a high correlation. Moreover, Foster, J. E. et al.^8^ showed no significant difference in cardiac axis during the cardiac cycle. Third, the electrophysiological axis also affects the myocardium volume and Purukinje network, which have great inter-individual variability^31^. The volunteers did not undergo echocardiography; thus, structural features, including LVH and myocardial volume, could not be evaluated. Fourth, the volunteers held their breath after the inhale during the CT scan, but not during the ECG recording. This may have potentially affected the results; however, previous studies have shown that the cardiac axis did not change with respiration^8^. Fifth, this volunteer study protocol did not include diseased patients. Further studies will be needed to assess the clinical implications.

## Conclusions

The QRS and anatomical axes (AV-apex axis and MV-apex axis) were significantly correlated in both supine and upright positions, while the angle of the AV-apex to the Z-axis was the most correlated. The anatomical axis moved in the dorsal and caudal directions from the supine to the upright position. The anatomical axis from the AV-apex to the Z-axis was determined based on age, body height, subcutaneous and visceral fat volumes (Fig. 2).

## Methods

This prospective study was approved by the institutional review board (KEIO UNIVERSITY SCHOOL OF MEDICINE AN ETHICAL COMMITTEE, Clinical Trail Number: UMIN000026586 [jRCTs032180267], first trial registration: 17/03/2017, first enrollment: 28/06/2017), and written informed consent was obtained from all participants. All methods were performed in accordance with relevant guidelines / regulations and the declaration of Helsinki. Healthy volunteers were recruited from a volunteer recruitment company between July 2017 and March 2019. Volunteers aged over 30 years were preferred to allow for the better understanding of the purpose of the study. Individuals with a history of hypertension, dyslipidemia, diabetes, or smoking, who had previously undergone cardiac surgery, or who were currently receiving treatment were excluded.

### Image acquisition

All volunteers underwent conventional 320-detector row CT (Aquilion ONE, Canon Medical Systems Corporation, Otawara, Japan) prospectively in the supine position. In addition, upright CT (prototype TSX-401R, Canon Medical Systems Corporation, Otawara, Japan) was performed in the standing position immediately after the conventional procedure. The upright and conventional supine CT scanners were adjacent to each other, and the two examinations were consecutively performed. The upright CT system was characterized by the up-and-down movements of a transverse 320 row-detector gantry (isotropic 0.5 mm in detector size), with a bore size of 780 mm, gantry rotation speed of 0.275 s, maximum vertical speed of 100 mm/s, and 1,200 view at optimal performance. Scanning was performed at 100 kVp and gantry rotation speed of 0.5 s in the helical scan mode (80-row detector), with a noise index of 24 and helical pitch of 0.8 for the body trunk from the level of the superior margin of the external acoustic meatus to the lowest position of the upright CT. Image reconstruction was performed using Adaptive Iterative Dose Reduction 3D (Canon Medical Systems Corporation, Otawara, Japan), which could reduce radiation dose.

### Image analysis

CT images were transferred to an off-line workstation [SYNAPSE VINCENT (FujiFilm, Tokyo, Japan) and Vitrea (Canon Medical Systems, Otawara, Japan)], and two blinded observers (T No and T Na) made multi-planner reconstruction images. On the multiplanar reconstruction (MPR) images, the anatomical long axis of the left ventricle was defined as extending from the center of the annulus of the mitral valve (MV) to the apex (blue and yellow plane). Other MPR images of the aorta were reconstructed on the perpendicular line to the ST junction plane, and the ostium of the aorta was defined as the annulus of the atrial valve (AV)^7,9,32^ (Supplemental Fig. 7, 8). The coordinates (X, Y, Z) of the apex [yellow dot: (X_apex_,Y_apex_,Z_apex_)] and annulus of the AV [blue dot: (X_AV_,Y_AV_,Z_AV_)] and MV [white dot: (X_MV_,Y_MV_,Z_MV_)] were measured.

The vectors of the AV-apex [(Xapex − X_AV_), (Yapex − Y_AV_), (Zapex − Z_AV_)] and MV-apex [(Xapex − X_MV_), (Yapex − Y_MV_), (Zapex − Z_MV_)] were calculated from the difference in the coordinates, and the 3D angles against X (1,0,0), Y (0,1,0), and Z axes (0,0,1) were calculated. For example, the angle between the AV-apex and X-axis was calculated as follows:

AV-apex to X-axis °: Degree (Arccos < (X_apex_ − X_AV_) × 1 + (Y_apex_ − Y_AV_) × 0 + (Z_apex_ − Z_AV_) × 0)/[{(X_apex_ − X_AV_)^2 + (Y_apex_ − Y_AV_)^2 + (Z_apex_ − Z_AV_)^2}^0.5 × {1^2 + 0^2 + 0^2}^0.5] >).

2D angles were also calculated against the X-axis in the XY plane, X-axis in the XZ plane, and Y-axis in the YZ plane.

Subcutaneous and visceral fat volumes at the belly button level on supine CT, which correlated with the total fat volume, were measured using a semi-automated algorithm of the workstation.

### Electrocardiography

Electrocardiography systems were utilized from September 2018 to March 2019 in the study period. After acquiring CT images, the male volunteers in this period underwent electrocardiography (ECG) test with the same electrocardiograms (ECG-2250, Nihon-Koden, Tokyo, Japan), which was programmed for a paper speed of 25 mm/s, gain of 5 and/or 10 mm/mV, and a low-frequency limit of 0.05 Hz following a recommendation by the American Heart Association to prevent ST-segment distortion^33^. Mason–Liker electrode placement for obtaining standard ECG. The electrical cardiac axis was automatically determined using an electrocardiography system with the I and II (Supplemental Fig. 7).

### Statistical analysis

The interobserver variability (NoT and NaT) of the AV-apex and MV-apex angles against all axes was determined using the Bland–Altman analysis and Pearson correlation coefficient.

Continuous data were tested for normality with Shapiro–Wilk test. The values of normal distribution were expressed mean ± one standard deviation and were compared between the two groups using paired t-test. Pearson’s correction coefficient test was used to assess the linear correlation between the two parameters. A two-sided *p* < 0.05 was considered statistically significant. A multivariate linear stepwise regression model was used to identify the relationship between the QRS axis or AV-apex to the Z-axis and anatomical parameters in both the supine and upright positions. All analyses were performed using SAS software (version 9.4; SAS Institute Inc., Cary, North Carolina).

### Supplementary Information

Supplementary Information.

## Data Availability

The datasets generated during and/or analyzed during the current study are available from the corresponding author upon reasonable request.

## Abbreviations

AV: Aortic valve
MV: Mitral valve
B.W.: Body weight
B.H.: Body height
B.M.I.: Body mass index
ECG: Electrocardiography

## References

[1] Ashnan R, Gardberg M, Byer E (1943). The normal human ventricular gradient. III. The relation between the anatomic and electrical axes. Am. Heart J..

[2] Hyman A, Failey RB, Ashman R (1948). A can the longitudinal anatomical axis of the ventricles be estimated from the electrocardiogram?. Am. Heart J..

[3] Fowler NO, Braunstein JR (1951). Anatomic and electrocardiographic position of the heart. Circulation.

[4] Dougherty JD (1970). The relation of the frontal QRS axis to the anatomic position of the heart. J. Electrocardiol..

[5] Meek WJ, Wilson A (1925). The effect of changes in position of the heart on the QRS complex of the electrocardiogram. Arch. Intern. Med..

[6] Engblom H, Hedström E, Palmer J, Wagner GS, Arheden H (2004). Determination of the left ventricular long-axis orientation from a single short-axis MR image: Relation to BMI and age. Clin. Physiol. Funct. Imaging.

[7] Engblom H (2005). The relationship between electrical axis by 12-lead electrocardiogram and anatomical axis of the heart by cardiac magnetic resonance in healthy subjects. Am. Heart J..

[8] Foster JE (2005). Determination of left ventricular long-axis orientation using MRI: changes during the respiratory and cardiac cycles in normal and diseased subjects. Clin. Physiol. Funct. Imaging.

[9] Sathananthan G (2015). Computed tomography-guided in vivo cardiac orientation and correlation with ECG in individuals without structural heart disease and in age-matched obese and older individuals. Clin. Anat..

[10] Jinzaki M (2020). Development of upright computed tomography with area detector for whole-body scans: Phantom study, efficacy on workflow, effect of gravity on human body, and potential clinical impact. Investig. Radiol..

[11] Yamada Y (2020). Differences in lung and lobe volumes between supine and standing positions scanned with conventional and newly developed 320-detector-row upright CT: Intra-individual comparison. Respiration.

[12] Yokoyama Y (2021). Effect of gravity on brain structure as indicated on upright computed tomography. Sci. Rep..

[13] Yagi F (2021). Three-dimensional evaluation of the coccyx movement between supine and standing positions using conventional and upright computed tomography imaging. Sci. Rep..

[14] Nakahara T (2021). Saphenous vein valve assessment utilizing upright CT to potentially improve graft assessment for bypass surgery. Sci. Rep..

[15] Goldberger, D. M. M. A. A. L. Electrocardiography. In *Braunwald's heart disease: A textbook of cardiovascular medicine 12 edn* (eds. Bonow Peter Libby, R. O. , Mann, D. L., Tomaselli, G. F., Bhatt, D. L., Solomon, S. D. & Braunwald, E.), Ch. 14, 141–174 (Elsevier, 2022).

[16] Jacobson, F. l. Chest Radiography in Cardiovascular Disease*.* In *Braunwald's heart disease: A textbook of cardiovascular medicine* 12 edn (eds. Bonow Peter Libby, R. O., Mann, D. L., Tomaselli, G. F., Bhatt, D. L., Solomon, S. D. & Braunwald, E) ch. 17, 268–276 (Elsevier, 2022).

[17] Dougherty JD (1970). Change in the frontal QRS axis with changes in the anatomic positions of the heart. J. Electrocardiol..

[18] Dougherty JD, Stoudt HW (1970). The relation of frontal QRS axis to age and body build. J. Electrocardiol..

[19] Tahara Y, Mizuno H, Ono A, Ishikawa K (1991). Evaluation of the electrocardiographic transitional zone by cardiac computed tomography. J. Electrocardiol..

[20] Stanley Nattel, G. F. T. Mechanisms of cardiac arrhythmias. In *Braunwald's heart disease: A textbook of cardiovascular medicine* 12edn (eds Bonow Peter Libby, R. O., Mann, D. L., Tomaselli, G. F., Bhatt, D. L., Solomon, S. D. & Braunwald, E) Ch. 62, 1163–1190 ( Elsevier, 2022).

[21] Rodriguez ER, Tan CD (2017). Structure and anatomy of the human pericardium. Prog. Cardiovasc. Dis..

[22] Martin M. Lewinter, P. C. C., Allan L. Klein. Pericardial Diseases. In *Braunwald's heart disease: A textbook of cardiovascular medicine* 12edn (ed Bonow Peter Libby, R. O., Mann, D. L., Tomaselli, G. F., Bhatt, D. L., Solomon, S. D., Braunwald, E.) Ch. 86, 1615–1634 (Elsevier, 2022).

[23] Boineau JP, Spach MS (1968). The relationship between the electrocardiogram and the electrical activity of the heart. J. Electrocardiol..

[24] Bachman S, Sparrow D, Smith LK (1981). Effect of aging on the electrocardiogram. Am. J. Cardiol..

[25] Rijnbeek PR (2014). Normal values of the electrocardiogram for ages 16–90 years. J. Electrocardiol..

[26] Zack PM, Wiens RD, Kennedy HL (1984). Left-axis deviation and adiposity: the United States Health and Nutrition Examination Survey. Am. J. Cardiol..

[27] Frank S, Colliver JA, Frank A (1986). The electrocardiogram in obesity: Statistical analysis of 1029 patients. J. Am. Coll. Cardiol..

[28] Bland EF, White PD (1931). The clikical significance of complete inversion of lead III of the human electrocardiogram. Am. Heart J..

[29] Simonson E, Keys A (1952). The effect of age and body weight on the electrocardiogram of healthy men. Circulation.

[30] Jones J, Srodulski ZM, Romisher S (1990). The aging electrocardiogram. Am. J. Emerg. Med..

[31] Massing GK, James TN (1976). Anatomical configuration of the His bundle and bundle branches in the human heart. Circulation.

[32] Mori S (2014). Association between the rotation and three-dimensional tortuosity of the proximal ascending aorta. Clin. Anat..

[33] Mirvis DM (1989). Instrumentation and practice standards for electrocardiographic monitoring in special care units. A report for health professionals by a task force of the Council on Clinical Cardiology American Heart Association. Circulation.

