# Supplementary material for: Anatomical cardiac and electrocardiographic axes correlate in both upright and supine positions: an upright/supine CT study
Source: Sci Rep. 2023 Oct 24;13:18170. doi: 10.1038/s41598-023-45528-y (PMC10598224; doi:10.1038/s41598-023-45528-y)

**Supplemental Table. 1** Volunteer characteristics

| | | | |
|---|---|---|---|
| Number | : | 41 | (all male) |
| Age (years-old) | : | 48.4 ± 12.0 | (range: 30-80) |
| B.H. (cm) | : | 171.6 ± 6.3 | (range:159.0-188.8) |
| B.W.(kg) | : | 70.0 ± 9.2 | (range: 52.0-94.0) |
| BMI | : | 23.8 ± 2.8 | (range: 18.6-31.5) |
| Body circumference (cm) | : | 87.6 ± 7.8 | (range: 70.2-107.0) |
| Subcutaneous fat volume ($cm^2$) | : | 122.5 ± 55.8 | (range: 21.6-297.0) |
| Visceral fat volume ($cm^2$) | : | 121.3 ± 60.0 | (range: 17.2-269.6) |

**Supplemental Table. 2** Multiple regression correlation for QRS axis

**Supine**

| | B | S.E. | Beta | t | p-value |
|---|---|---|---|---|---|
| Intercept | 109.5 | 10.1 | 0 | 10.84 | <0.0001 |
| AV-apex to the Z axis | - 0.7 | 0.2 | - 0.51 | - 3.75 | 0.0006 |

**Upright**

| | B | S.E. | Beta | t | p-value |
|---|---|---|---|---|---|
| Intercept | 121.7 | 20.7 | 0 | 5.88 | <0.0001 |
| AV-apex to the Z axis | - 3.4 | 1.2 | - 1.29 | - 2.81 | 0.0079 |
| MV-apex to the Z-axis | 2.2 | 1.1 | 0.91 | 1.97 | 0.0562 |

**Supplemental Table. 3** The partial correlation between QRS axis and parameters controlling the angle of AV-apex to the Z axis.

| Person's R | Supine | | Upright | |
|---|---|---|---|---|
| Age (years-old) | - 0.19 | (0.2394) | - 0.19 | (0.2409) |
| B.H. (cm) | - 0.36 | (0.0206*) | - 0.43 | (0.0055*) |
| B.W. (kg) | - 0.12 | (0.4547) | - 0.30 | (0.0631) |
| BMI | 0.08 | (0.6214) | - 0.09 | (0.5931) |
| Body circumference (cm) | - 0.15 | (0.3689) | - 0.34 | (0.0329*) |
| Subcutaneous fat volume ($cm^2$) | - 0.00 | (0.9851) | - 0.13 | (0.4219) |
| Visceral fat volume ($cm^2$) | - 0.04 | (0.7953) | - 0.13 | (0.4266) |

**Supplemental Table. 4** The correlation between the angle of AV-apex to the Z axis and volunteer characteristics' parameters (*: $p<0.05$)

| Person's R | Supine | Upright |
|---|---|---|
| Age (years-old) | 0.56 (0.0001*) | 0.57 (<0.0001*) |
| B.H. (cm) | - 0.31 (0.0479*) | - 0.33 (0.0366*) |
| B.W. (kg) | 0.24 (0.1230) | 0.27 (0.0846) |
| BMI | 0.47 (0.0018*) | 0.51 (0.0006*) |
| Body circumference (cm) | 0.54 (0.0002*) | 0.56 (0.0001*) |
| Subcutaneous fat volume ($cm^2$) | 0.54 (0.0003*) | 0.54 (0.0003*) |
| Visceral fat volume ($cm^2$) | 0.60 (<0.0001*) | 0.64 (<0.0001*) |

**Supplemental Table. 5** Multiple regression correlation for the angle of AV-apex to the Z axis

**Supine**

| | B | S.E. | Beta | t | p-value |
|---|---|---|---|---|---|
| Intercept | 64.28 | 31.17 | 0 | 2.06 | 0.0465 |
| Age (years-old) | 0.38 | 0.09 | 0.44 | 4.03 | 0.0003 |
| B.H. (cm) | - 0.26 | 0.17 | - 0.15 | - 1.48 | 0.1475 |
| Subcutaneous fat volume ($cm^2$) | 0.07 | 0.02 | 0.36 | 2.80 | 0.0081 |
| Visceral fat volume ($cm^2$) | 0.04 | 0.02 | 0.24 | 1.84 | 0.0740 |

**Upright**

| | B | S.E. | Beta | t | p-value |
|---|---|---|---|---|---|
| Intercept | 65.10 | 24.11 | 0 | 2.70 | 0.0105 |
| Age (years-old) | 0.31 | 0.07 | 0.43 | 4.17 | 0.0002 |
| B.H. (cm) | - 0.23 | 0.13 | - 0.17 | - 1.74 | 0.0901 |
| Subcutaneous fat volume ($cm^2$) | 0.05 | 0.02 | 0.31 | 2.62 | 0.0126 |
| Visceral fat volume ($cm^2$) | 0.05 | 0.02 | 0.32 | 2.55 | 0.0150 |

**Supplemental Figure legends**

**Supplemental Figure. 1 The angle of AV-apex to X axis demonstrated excellent inter-observer reproducibility in both supine and upright position.**

To evaluate the reproducibility of the angle of AV-apex to X axis, inter-observer variability is assessed by Bland-Altman analysis and Spearman's R. (A, B) Inter observer viability in supine position. Two observers (T.No. and T.Na.) assessed the same 41 patients independently. The Bland-Altman analysis shows agreement of inter observer reproducibility (A) and the Spearman's R shows high correlation ($R= 0.97$, $p<0.0001$) (B). (C, D) Inter observer viability in upright position. Two observers (T.No. and T.Na.) assessed the same 41 patients independently. The Bland-Altman analysis shows agreement of inter observer reproducibility (C) and the Spearman's R shows high correlation ($R= 0.98$, $p<0.0001$) (D).

**Supplemental Figure. 2 The angle of AV-apex to Y axis demonstrated excellent inter-observer reproducibility in both supine and upright position.**

To evaluate the reproducibility of the angle of AV-apex to Y axis, inter-observer variability is assessed by Bland-Altman analysis and Spearman's R. (A, B) Inter observer viability in supine position. Two observers (T.No. and T.Na.) assessed the same 41 patients independently. The Bland-Altman analysis shows agreement of inter observer reproducibility (A) and the

Spearman's R shows high correlation (R= 0.97, p<0.0001) (B). (C, D) Inter observer viability in upright position. Two observers (T.No. and T.Na.) assessed the same 41 patients independently. The Bland-Altman analysis shows agreement of inter observer reproducibility (C) and the Spearman's R shows high correlation (R= 0.97, p<0.0001) (D).

**Supplemental Figure. 3 The angle of AV-apex to Z axis demonstrated excellent inter-observer reproducibility in both supine and upright position.**

To evaluate the reproducibility of the angle of AV-apex to Z axis, inter-observer variability is assessed by Bland-Altman analysis and Spearman's R. (A, B) Inter observer viability in supine position. Two observers (T.No. and T.Na.) assessed the same 41 patients independently. The Bland-Altman analysis shows agreement of inter observer reproducibility (A) and the Spearman's R shows high correlation (R= 0.99, p<0.0001) (B). (C, D) Inter observer viability in upright position. Two observers (T.No. and T.Na.) assessed the same 41 patients independently. The Bland-Altman analysis shows agreement of inter observer reproducibility (C) and the Spearman's R shows high correlation (R= 0.98, p<0.0001) (D).

**Supplemental Figure. 4 The angle of MV-apex to X axis demonstrated excellent inter-observer reproducibility in both supine and upright position.**

To evaluate the reproducibility of the angle of MV-apex to X axis, inter-observer variability is assessed by Bland-Altman analysis and Spearman's R. (A, B) Inter observer viability in supine position. Two observers (T.No. and T.Na.) assessed the same 41 patients independently. The Bland-Altman analysis shows agreement of inter observer reproducibility (A) and the Spearman's R shows high correlation (R= 0.95, p<0.0001) (B). (C, D) Inter observer viability in upright position. Two observers (T.No. and T.Na.) assessed the same 41 patients independently. The Bland-Altman analysis shows agreement of inter observer reproducibility (C) and the Spearman's R shows high correlation (R= 0.97, p<0.0001) (D).

**Supplemental Figure. 5 The angle of MV-apex to Y axis demonstrated excellent inter-observer reproducibility in both supine and upright position.**

To evaluate the reproducibility of the angle of MV-apex to Y axis, inter-observer variability is assessed by Bland-Altman analysis and Spearman's R. (A, B) Inter observer viability in supine position. Two observers (T.No. and T.Na.) assessed the same 41 patients independently. The Bland-Altman analysis shows agreement of inter observer reproducibility (A) and the Spearman's R shows high correlation (R= 0.97, p<0.0001) (B). (C, D) Inter observer viability in upright position. Two observers (T.No. and T.Na.) assessed the same 41 patients independently. The Bland-Altman analysis shows agreement of inter observer reproducibility (C) and the

Spearman's R shows high correlation (R= 0.95, p<0.0001) (D).

**Supplemental Figure. 6 The angle of MV-apex to Z axis demonstrated excellent inter-observer reproducibility in both supine and upright position.**

To evaluate the reproducibility of the angle of MV-apex to Z axis, inter-observer variability is assessed by Bland-Altman analysis and Spearman's R. (A, B) Inter observer viability in supine position. Two observers (T.No. and T.Na.) assessed the same 41 patients independently. The Bland-Altman analysis shows agreement of inter observer reproducibility (A) and the Spearman's R shows high correlation (R= 0.99, p<0.0001) (B). (C, D) Inter observer viability in upright position. Two observers (T.No. and T.Na.) assessed the same 41 patients independently. The Bland-Altman analysis shows agreement of inter observer reproducibility (C) and the Spearman's R shows high correlation (R= 0.97, p<0.0001) (D).

**Supplemental Figure. 7** A schema of definition of parameters.

The coordinates of the apex, center of the aortic valve (AV), and mitral valve (MV) were set; the vectors were calculated from the coordinates, and the angles of the vectors were calculated in both two (2D) and three (3D) dimensions.

The electrical cardiac axis was automatically determined using an electrocardiography system with the I and augmented Vector Foot (aVF).

**Supplemental Figure. 8** Definition of aortic valve (AV), mitral valve (MV), and apex on computed tomography images.

The multiplanar reconstruction (MPR) images were reconstructed using an offline workstation. (A) Long axis of the left ventricle. Referring to the orthogonal sections, the anatomical long axis of the left ventricle was defined as extending from the center of the annulus of the MV to the apex (intersection line of the blue and yellow planes). (B) Orthogonal section of the ST-junction. Another MPR image of the aorta was reconstructed on a line perpendicular to the ST-junction plane. Referring to the orthogonal sections, the center of the aortic root was expressed as the intersection line of the blue and yellow planes. The ostium of the aortic root was defined as the annulus of the AV.

**Supplemental Figure. 1**

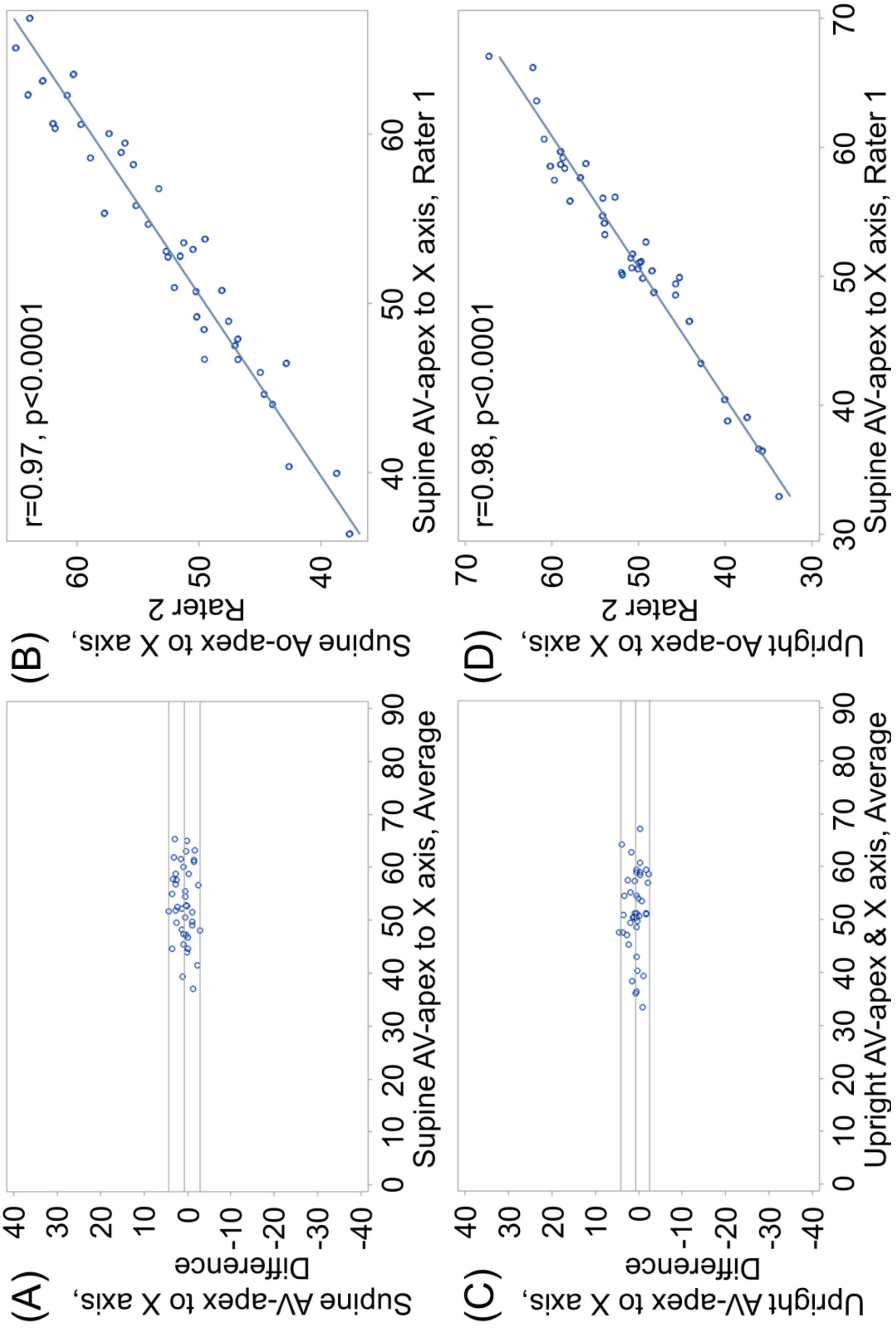

**Supplemental Figure. 2**

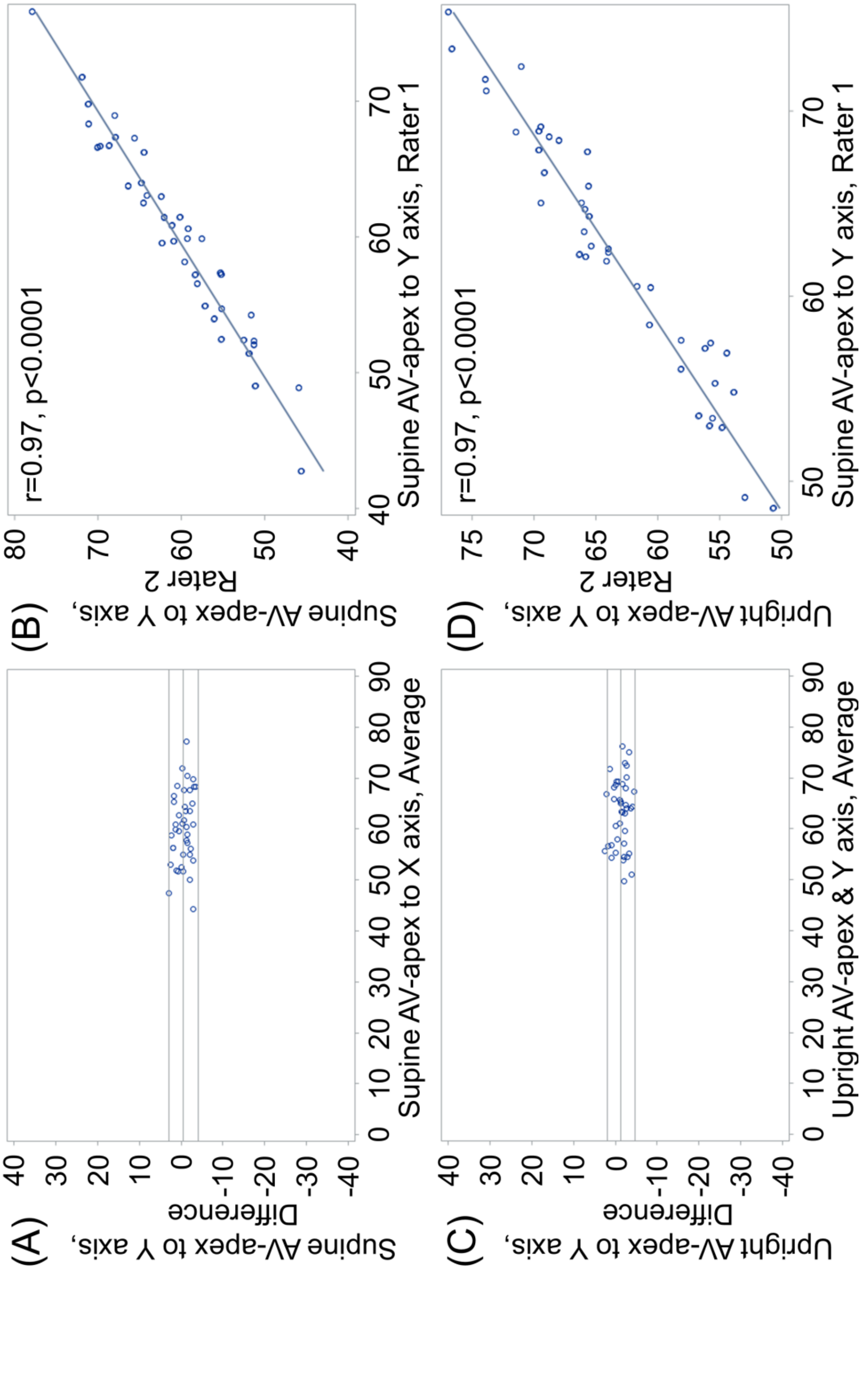

**Supplemental Figure. 3**

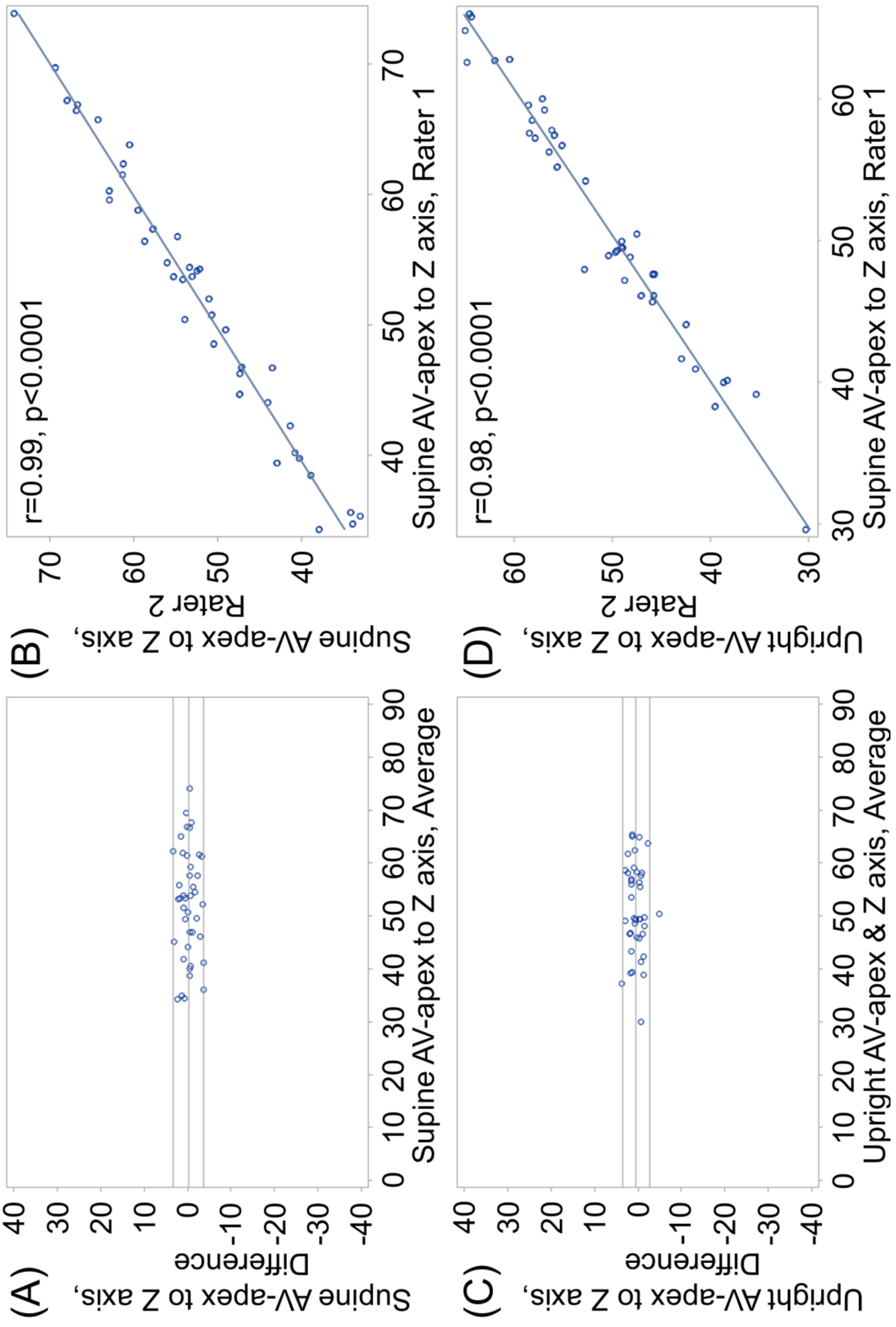

**Supplemental Figure. 4**

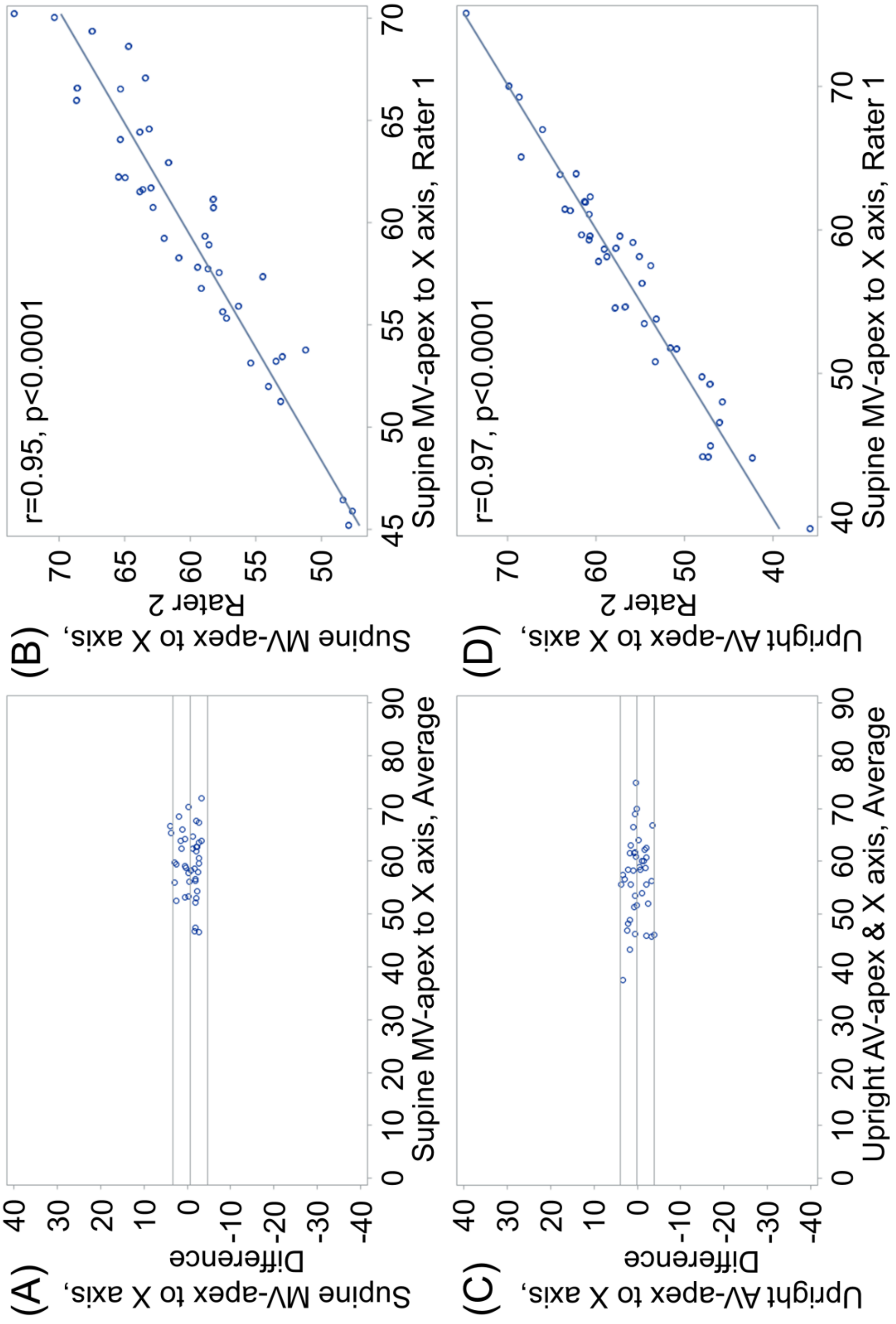

**Supplemental Figure. 5**

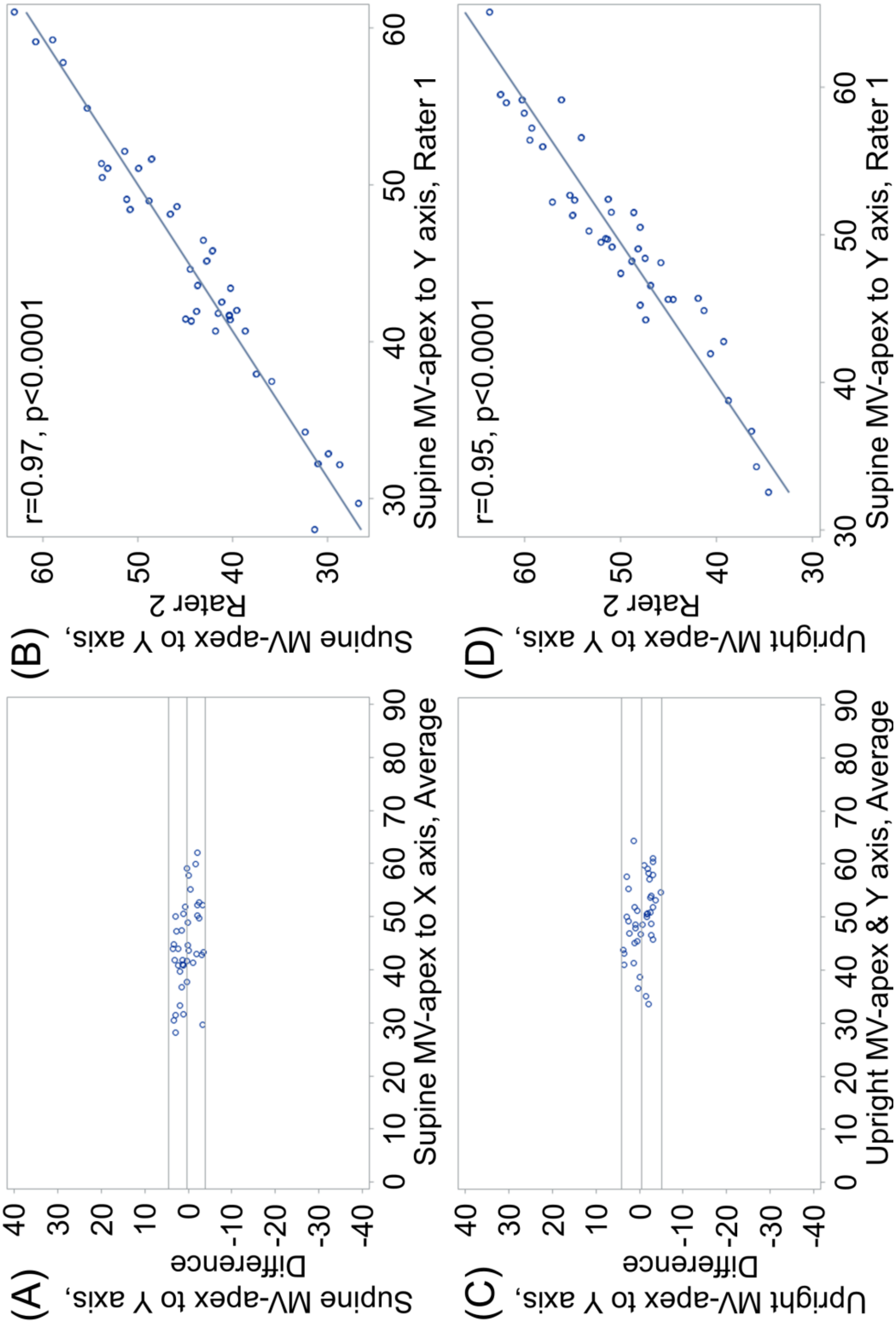

**Supplemental Figure. 6**

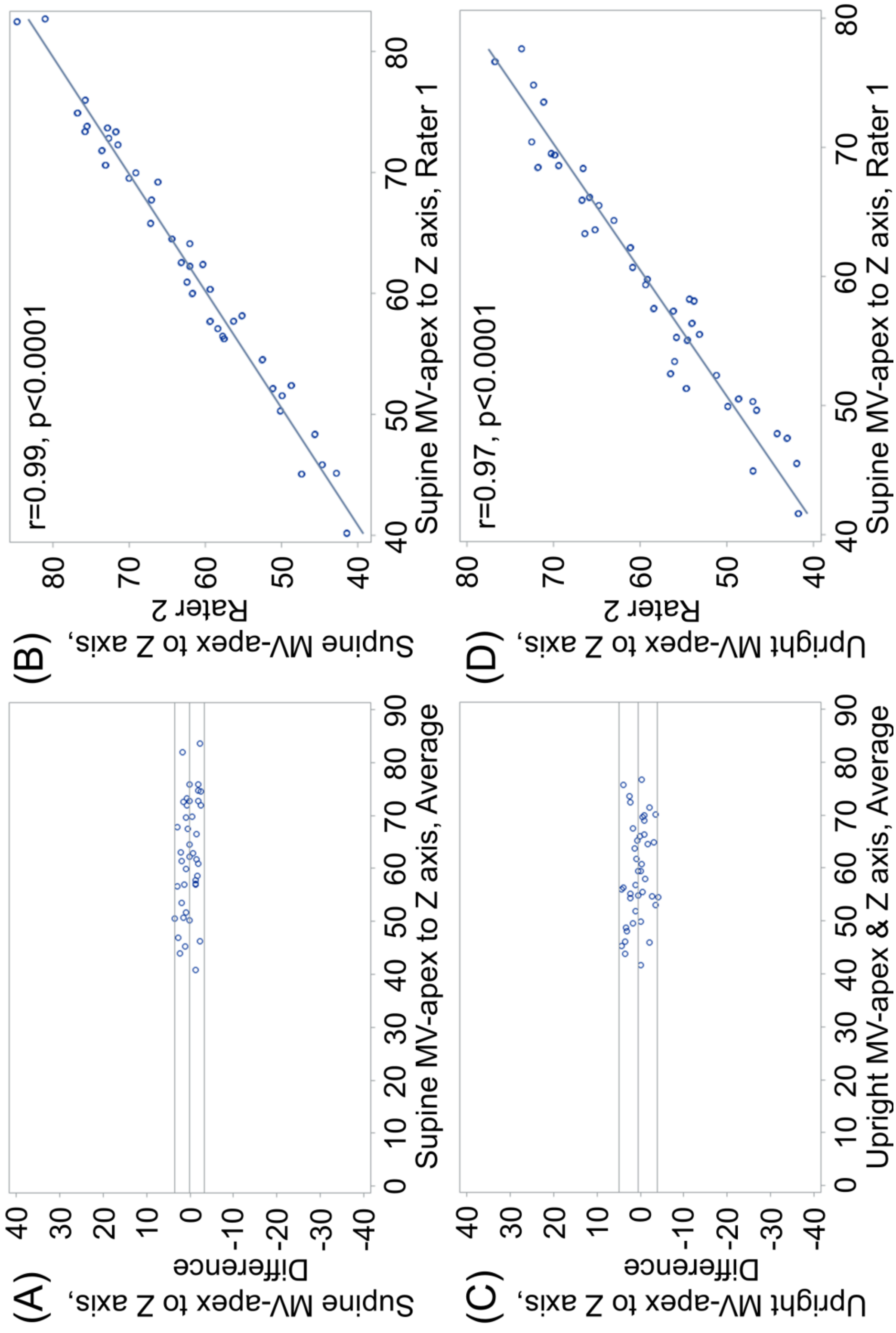

**Supplemental Figure.7**

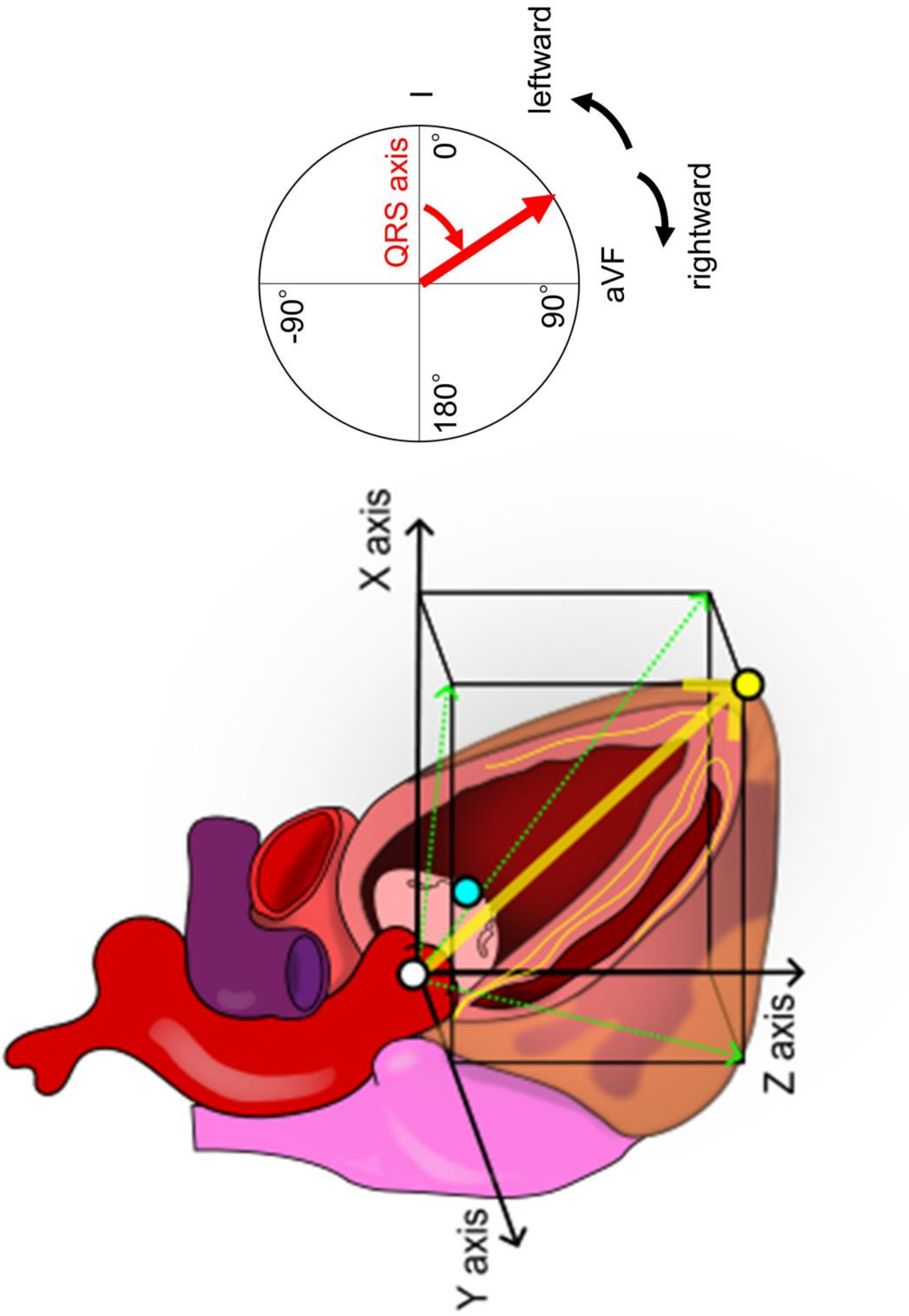

**Supplemental Figure.8**

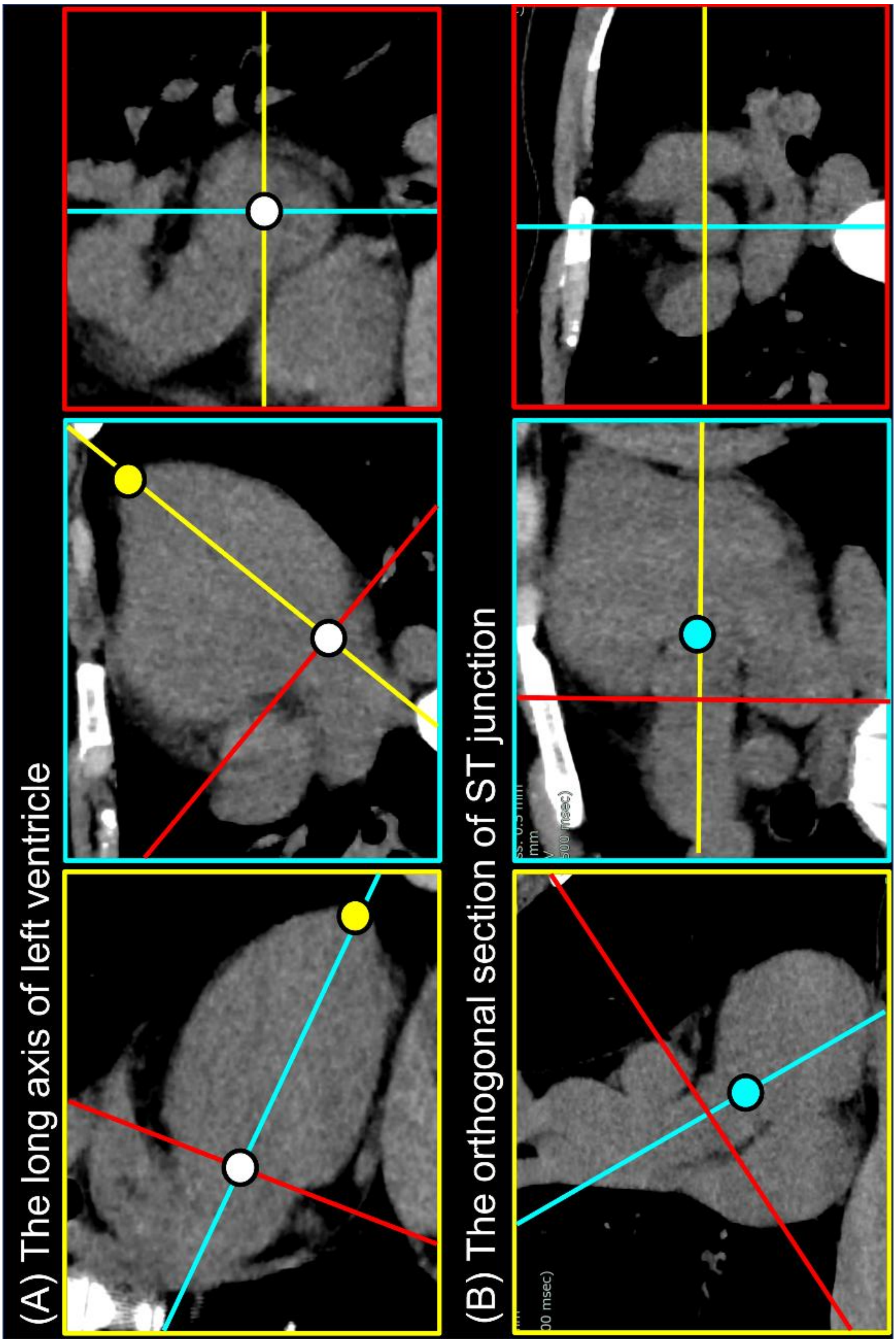

Supplement: Supplementary file 1 — Supplementary Information. [file 41598_2023_45528_MOESM1_ESM.pdf]
